# Endosomal trafficking inhibitor EGA can control TLR7-mediated IFNα expression by human plasmacytoid dendritic cells

**DOI:** 10.3389/fimmu.2023.1202197

**Published:** 2023-11-24

**Authors:** Matthew J. Wiest, Laurie Baert, Chao Gu, Kevin M. Gayler, Hyoungjun Ham, Laurent Gorvel, Mira T. Keddis, Leroy W. Griffing, HyeMee Joo, Jean-Pierre Gorvel, Daniel D. Billadeau, Robert R. Kane, SangKon Oh

**Affiliations:** ^1^ Department of Immunology, Mayo Clinic, Scottsdale, AZ, United States; ^2^ Department of Chemistry and Biochemistry, Baylor University, Waco, TX, United States; ^3^ Department of Immunology, Mayo Clinic, Rochester, MN, United States; ^4^ CRCM, Aix Marseille Universite, INSERM, Marseille, France; ^5^ Department of Nephrology, Mayo Clinic, Scottsdale, AZ, United States; ^6^ Department of Rheumatology, Mayo Clinic, Scottsdale, AZ, United States; ^7^ Aix Marseille Univ, CNRS, INSERM, CIML, Marseille, France

**Keywords:** EGA, endosome trafficking, nucleic acid, innate immunity, plasmacytoid dendritic cells, TLR7, type 1 interferon, proinflammatory cytokine

## Abstract

Plasmacytoid dendritic cells (pDC) are the major producer of type 1 IFN in response to TLR7 agonists. Aberrant TLR7 activation and type 1 IFN expression by pDCs are linked to the pathogenesis of certain types of autoimmune diseases, including systemic lupus erythematosus (SLE). This study investigated the underlying mechanisms for TLR7-mediated cytokine expression by pDCs using a late endosome trafficking inhibitor, EGA (4-bromobenzaldehyde *N*-(2,6-dimethylphenyl) semicarbazone). We found that EGA treatment decreased IFNα expression by pDCs stimulated with imiquimod (R837), single-stranded RNA40, and influenza virus. EGA also decreased TNFα expression and secretion by R837-stimulated pDCs. Mechanistically, EGA treatment decreased phosphorylation of IKKα/β, STAT1, and p38, and prolonged degradation of IκBα. Furthermore, EGA treatment decreased the colocalization of 3F, a substituted adenine TLR7 agonist, with LAMP1^+^ compartments in pDCs. EGA was also capable of diminishing IFNα expression by SLE pDCs treated with R837 or live PR8/A/34 influenza viruses. Therefore, we concluded that trafficking of TLR7 agonists to LAMP1^+^ compartments is important for IFNα expression by pDCs. Data from this study support additional examinations of the potential benefits of EGA in treating type 1 IFN-associated inflammatory diseases in the future.

## 1 Introduction

Plasmacytoid dendritic cells (pDCs) are the major producer of type 1 interferon (IFN) in response to nucleic acids derived from self (1–4) and non-self origin (5–8). Type 1 IFN produced by pDCs has beneficial roles in host immunity to viral infections (9, 10), but it is also implicated in the pathogenesis of certain types of autoimmune diseases, including systemic lupus erythematosus (SLE) (11, 12), especially through an aberrant TLR7 activation (13, 14). TLR7-activated pDCs can also produce proinflammatory cytokines, including TNFα and IL-6. It is thus important to understand the underlying mechanisms for the TLR7-mediated cytokine expression by pDCs. In addition, finding strategies that can manipulate cytokine expression by TLR7-activated pDCs, may also help control certain inflammatory conditions, including SLE, and excessive innate cytokine response during certain viral infections (15, 16).

It was previously shown that small molecule TLR7 agonists do not localize to early endosomes, yet they can induce IFNα production by human pDCs (17), which is in contrast with the previously proposed model that, in human pDCs, TLR9 signaling from early endosomes (EEA1^+^) leads to IFNα production, whereas signaling from late endosomes (LAMP1^+^) leads to proinflammatory cytokine production (18). However, Sasai et al. (19) reported that signals for TLR9-mediated type 1 IFN expression are induced in a LAMP2^+^ lysosomal-related organelle (LRO), whereas VAMP3^+^ NF-κB endosomes are responsible for proinflammatory cytokine expression in mouse bone marrow-derived pDCs and macrophages. Another recent study (20) also reported that an inducible VAMP3^+^LAMP2^+^LAMP1^−^ endolysosome compartment exists in human pDCs from which TLR9 activation triggers type 1 interferon expression, whereas TLR9 trafficking to LAMP1^+^ late endosomes leads to NF-κB activation and TNF production. Interestingly, however, mice with genetic ablation of genes responsible for LRO formation showed diminished expression of both type 1 IFN and proinflammatory cytokines by bone marrow-derived murine pDCs in response to TLR7/8 ligand imidazoquinoline stimulation (21). In addition, TLR7 agonists are also known to passively diffuse into pDCs and then accumulate in an active process dependent on the action of v-ATPase in LAMP-1^+^ CD63^+^ MHCII^+^ endo-lysosomes (17). Although data from these studies (17–21) significantly advanced our understanding of human pDC cytokine expression in response to nucleic acids, the underlying mechanisms for the TLR7-mediated cytokine expression by pDCs remain to be fully investigated. Notably, however, data from previous studies (17–21) suggest that the transition between early/recycling to endo-lysosomes might be a key determinant for the cytokine response, type 1 IFN or proinflammatory cytokines, produced by pDCs stimulated with different types of nucleic acids.

EGA, 4-bromobenzaldehyde *N*-(2,6-dimethylphenyl) semicarbazone, was first reported as an inhibitor of anthrax lethal toxin-induced pyroptosis in macrophages (22). EGA is known to block the trafficking from EEA1^+^ endosomes to LAMP1^+^ endosome/lysosome without affecting recycling endosome trafficking. EGA did not alter the acidification of endo-lysosomes, phago-lysosomal trafficking, and phagosome permeabilization (22).

Therefore, we hypothesized that inhibition of endosomal trafficking from the early/recycling endosome to the endo-lysosome compartment with EGA could alter the cytokine responses by pDCs stimulated with TLR7 agonists. We tested this hypothesis by assessing cytokine expression by pDCs in response to imiquimod (R837). Mechanistic insight into the EGA-mediated modulation of cytokine responses by pDCs was also investigated. Using fluorochrome conjugated 3F (23), a substituted adenine TLR7 ligand, we also investigated its endo-lysosomal trafficking in the presence and absence of EGA, in comparison with phosphatidylinositol 3-phosphate 5-kinase (PIKfyve) inhibitor. Lastly, we tested the effects of EGA on the cytokine expression by pDCs and monocytes/myeloid DCs (mDCs) isolated from the blood of adult SLE patients. Data from this study suggest that EGA can control IFNα and TNFα expression by pDCs stimulated via TLR7.

## 2 Materials and methods

### 2.1 pDC isolation and culture

Buffy coats (Oklahoma Blood Institute) and apheresis cones from Mayo Clinic were utilized as blood sources. PBMCs were collected from the blood by density gradient centrifugation utilizing Ficoll-Paque Plus (GE Healthcare). DCs were enriched from PBMCs with Pan-DC Enrichment Kit (Stemcell Technologies). pDCs (Lin-1^-^HLA-DR^+^ CD123^+^CD11c^-^) were sorted on a FACS Aria II (BD Biosciences). Purity after sorting was above 98%. pDCs were washed with complete RPMI (cRPMI) and resuspended in cRPMI/10% FBS (Gemini Bio-Products) containing 5 ng/mL IL-3 (R&D Systems). Complete RPMI consisted of RPMI1640 containing L-glutamine (ThermoFisher) supplemented with MEM non-essential amino acids, sodium pyruvate (Sigma Aldrich), and penicillin/streptomycin (ThermoFisher).

EGA (Cayman Chemical or Sigma Aldrich) and YM201636 (Cayman Chemical) were each dissolved in dimethyl sulfoxide (DMSO) (ThermoFisher), aliquoted, and frozen at -30°C. Fresh aliquots were utilized for each experiment. Cells were pre-incubated with 1μM YM201636, 20 μM EGA, or vehicle for 15-30 minutes at 37 °C. Approximately 5×10^4^ cells were stimulated with 5 μg/mL R837, 2 μg/mL R848 (Invivogen), 2 μg/mL ssRNA40 (Miltenyi Biotec), or 2 multiplicity of infection (MOI) live influenza A/PR8/34 (kindly provided by Dr. Adolfo Garcia-Sastre, Mount Sinai, NY). Complexes of ssRNA40 with 10 μg/mL 1,2-Dioleoyl-3-trimethylammonium propane (DOTAP Transfection Reagent; Santa Cruz Biotechnology) were prepared in LAL water (Invivogen) at least 15 minutes prior to be used. After 2 hours, GolgiPlug (BD Biosciences) was added according to the manufacturer’s instructions. Additional 3 hours later, cells were washed with 2 mM EDTA/DPBS (ThermoFisher), stained for viability, fixed, permeabilized with Cytofix/CytoPerm (BD Biosciences), and stained for intracellular cytokine expression in PermWash solution (BD Biosciences). Cells were also stained for surface TNFα on pDCs.

### 2.2 Flow cytometry and antibodies

**Supplementary Table 1** lists the antibodies utilized for FACS analyses in this study. Cells were run on a FACS Celesta (BD Biosciences) or Cytek Aurora (Cytek Biosciences). Flow cytometry data were analyzed utilizing FlowJo v10 (FlowJo). Intracellular and surface cytokine staining gating was based on the examination of cytokine staining for unstimulated cells and isotype control antibody staining.

### 2.3 Antibody-coated bead-based cytokine assay

Bead-based Luminex kits assessing human IFNα and TNFα (ThermoFisher) were utilized for measuring cytokine concentrations in supernatants from overnight stimulated pDCs. Beads were assessed on a Luminex 200 machine and the Xponent 3.1 software (Luminex) was utilized to analyze the data.

### 2.4 Western blot analysis

FACS-sorted pDCs were extensively washed and rested for at least one hour in cRPMI containing IL-3 (5 ng/mL) at 37 °C. pDCs were then treated with indicated inhibitors at 37 °C for 30 minutes. Approximately 1×10^5^ - 1.4×10^5^ pDCs/well in 96-well plates were stimulated with 5 μg/mL R837 for the indicated time frame upon cell lysates were harvested in RIPA buffer (ThermoFisher) supplemented with HALT™ protease and phosphatase inhibitor cocktail (ThermoFisher). Cell lysates were reduced, ran on Novex 4-20% Tris-Glycine gels (Invitrogen), and transferred to polyvinylidene difluoride (PVDF) membranes (Bio-Rad), as previously described (24). Protein normalization was performed utilizing a No-Stain Protein labeling reagent (ThermoFisher) according to the manufacturer’s instructions. Antibodies utilized for immunoblotting are listed in **Supplementary Table 2**. PageRuler prestained protein ladder (ThermoFisher, Catalog 26616) was used as a reference for protein size. Images were acquired on a ChemiDoc MP (Bio-Rad, CA) and analyzed in Image Lab V6.0 (Bio-Rad). Protein intensity was normalized either to total reference protein or total protein concentration. Relative intensity was obtained by comparison with vehicle-treated group at time zero.

### 2.5 Synthesis and characterization of 3F-AF488

3F compound has previously been characterized as a TLR7 agonist with similar efficacy to the imidazoquinoline, resiquimod (R848) (23). 3F and AF488 NHS (lumiprobe) were combined in equimolar concentrations in DMSO-D_6_ (Cambridge Isotopes). TEA (Sigma Aldrich) was added, and the mixture was placed at 25°C for 16 hours. The reaction product was purified into precipitate form with washing of ethyl acetate to yield a red solid which was dissolved in DMSO. Reactions were monitored utilizing NMR and Thermo Scientific Dionex Ultimate 3000 HPLC equipped with an Eclipse Plus C18 3.5 mM, 3.0 x100 mm column using a 5:95 to 95:5 H_2_O + 0.1% Formic Acid : Acetonitrile gradient.

HEK Blue hTLR7 cell line (Invivogen) and the parental cell line (Null-1k) were used to examine the ability of 3F-AF488 to activate cells via TLR7. Equivalent numbers of HEK Blue hTLR7 cells were stimulated with 10 μM 3F and 3F-AF488, and vehicle for 15 hours in HEK Blue detection media (Invivogen). NF-κB activity was measured by assessing the optical density at 620-655 nm using a SpectraMax 12 (Molecular Device) and analyzed with SoftMaxPro V5 software (Molecular Device).

### 2.6 Quantitative real-time PCR

Total RNA was extracted from cell lysates using protocol from the RNAqueous™ Micro Total RNA Isolation Kit (ThermoFisher). 200 ng of input total RNA was used to generate cDNAs using iScript Reverse Transcription Supermix for RT-PCR (Biorad) by following manufacturer’s protocol. The primer sequences used for human S18 were forward 5’-TGCCATCACTGCCATTAAGG-3’ and reverse 5’-TGCTTTCCTCAACACCACATG-3’, for human TNFα forward 5’- CTCTTCTGCCTGCTGCACTTTG-3’ and reverse 5’-ATGGGCTACAGGCTTGTCACTC-3’ and for human IFNα forward 5’-CCAGTTCCAGAAGGCTCCAG-3’ and reverse 5’-TCCTCCTGCATCACACAGGC-3’. SYBR green-based qPCRs with 2µl of cDNA were ran on a Roche LightCycler 480 instrument (Roche). The reactions were performed in 20 µl as follows: 50°C for 2 mins, 95°C for 2 mins, followed by 40 cycles of 95°C for 15 seconds, 52°C for 15 seconds and 72°C for 1mins. Results were analyzed using the 2-ΔΔ Ct method and normalized to the corresponding level of the housekeeping gene S18.

### 2.7 Confocal microscopy

pDCs were enriched utilizing a human plasmacytoid DC enrichment kit (Stemcell Technologies). Enriched cells were assessed for purity utilizing the same surface markers for sorting. After three consecutive enrichments, pDC purity was above 92 - 97%. pDCs were pre-incubated with inhibitors for 1 hour at 37 °C before the addition of 20 μM 3F-AF488. Cells were affixed to coverslips after 1, 2, and 5 hours of stimulation and stained as previously described (25). Briefly, pDCs were attached to poly-D-lysine (35 μg/mL; ThermoFisher)-coated coverslips, fixed with ice-cold 4% paraformaldehyde (Electron Microscopy Sciences), permeabilized with 0.15% Triton™ X-100 Surfactant-Amps (ThermoFisher), and then washed extensively. Cells were blocked and stained in a saline blocking buffer containing 5% goat serum (Invitrogen), 1% glycerol (Sigma-Aldrich), and 0.1% bovine serum albumin (MP Biomedicals). After blocking for 1 hour, cells were incubated with primary and secondary antibodies in a blocking buffer with extensive washing between steps. Cells were stained with antibodies listed in **Supplementary Table 3**. Cells were counterstained with Hoechst 33342 (ThermoFisher) according to the manufacturer’s instructions and washed. Coverslips were mounted on slides in Prolong Gold Antifade Mounting reagent (ThermoFisher). Slides were imaged on an LSM 800 Confocal Microscope (Zeiss) utilizing a 63x/1.4N.A objective. Identical acquisition settings were used for all experimental samples and images were scanned in frame mode to avoid any crosstalk between fluorophore signals.

### 2.8 Image processing

Cell images were processed via Airyscan in the Zen software module (Zeiss). Images were further processed in the open-source software Fiji (26). Baseline intensity thresholds for analysis were defined by cellular autofluorescence and isotype antibody intensity staining for each experiment. Manders’ coefficients were determined utilizing an in-house customized plugin coded for thresholding of 3F signal and region of interest selection (27).

### 2.9 PBMC isolation and culture

SLE patients (**Supplementary Table 4**) were recruited at Mayo Clinic under a protocol approved by the institutional review board. Patients provided informed consent in accordance with the Declaration of Helsinki. PBMCs were isolated from the whole blood of SLE patients. PBMCs were washed in cRPMI and resuspended in cRPMI/10% FBS (Gemini Bio-Products). PBMCs were pre-incubated with 20 μM EGA or vehicle for 30 minutes at 37 °C. Approximately 8×10^5^ - 1×10^6^ PBMCs per well in 96-well plates were stimulated with 5 μg/mL R837 or 2 MOI live influenza virus A/PR8/34. After 2 hours incubation, GolgiPlug (BD Biosciences) was added according to the manufacturer’s instructions. After an additional 3 hours of incubation, PBMCs were stained for viability and surface markers, fixed and permeabilized with Cytofix/CytoPerm (BD Biosciences), and stained for intracellular cytokine expression. Cells were run on FACS Fortessa (BD Biosciences).

### 2.10 Data and statistical analysis

Graphs with error bars represent mean ± SD (or SEM). Data were analyzed in Prism 7.0 (GraphPad) utilizing one-way analysis of variance (ANOVA), and two-way ANOVA with Tukey, Sidak, or t-test. Significance was set at p<0.05.

## 3 Results

### 3.1 EGA alters cytokine expression by R837-pDCs

**Figure 1:**
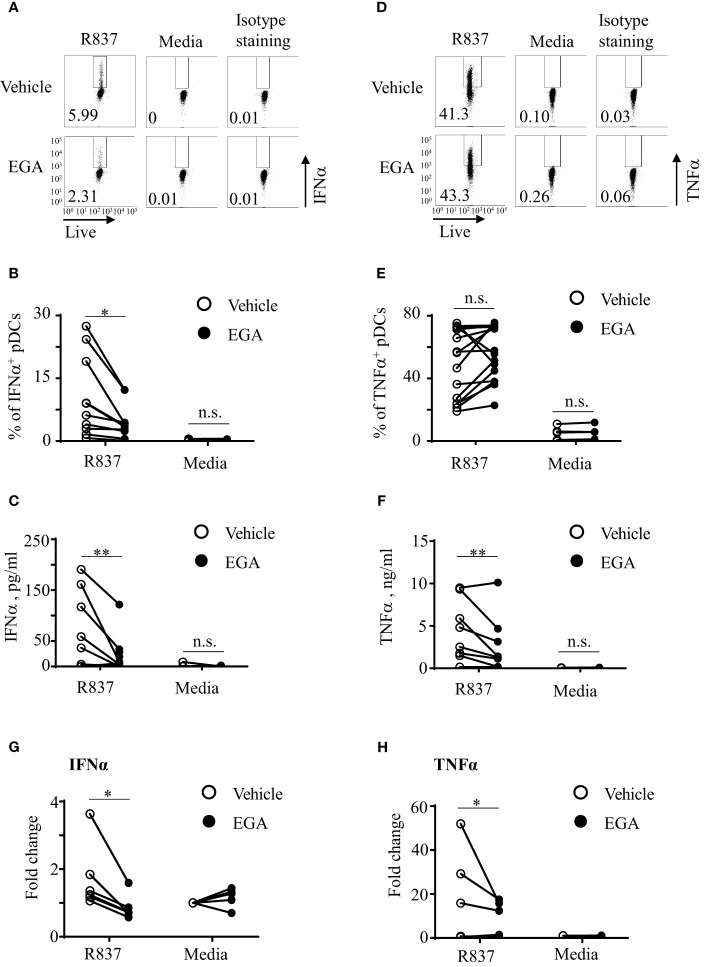
EGA diminishes IFNα and TNFα secretion by R837-stimulated pDCs. FACS-sorted pDCs were pre-incubated for 30 mins with 20 μM EGA or vehicle. pDCs were then stimulated with 5 μg/mL R837 for 5 hours and stained for intracellular IFNα expression. Representative FACS plots **(A)**. Summarized data of the frequency of intracellular IFNα^+^ pDCs **(B)**. After overnight culture, the amount of IFNα in culture supernatants was measured by bead-based cytokine assays **(C)**. Representative FACS plots of intracellular TNFα expression **(D)** and summarized data **(E)**. The amount of TNFα in culture supernatants **(F)**. Both IFNα **(G)** and TNFα **(H)** transcripts were measured by qRT-PCR. In **(B–G, E–H)**, individual lines indicate data generated with pDCs from different subjects. Data analyzed by paired t-test. *p < 0.05, **p < 0.01, and n.s., not significant, for the comparison between groups.

We began by assessing how EGA, a late endosomal trafficking inhibitor, would affect pDC responses to R837, a TLR7-specific imidazoquinoline. As shown in **Figure 1A**, EGA treatment decreased the frequency of IFNα^+^ pDCs when pDCs were stimulated with R837. Summarized data generated with pDCs of 10 healthy donors are presented in **Figure 1B**. EGA treatment also reduced the amount of IFNα secreted by R837-stimulated pDCs (**Figure 1C**).

Interestingly, however, the effects of EGA treatment on TNFα expression by R837-stimulated pDCs were variable among healthy donors especially when the frequency of TNFα^+^ pDCs was assessed by intracellular TNFα staining that could detect both surface and intracellular TNFα (**Figures 1D, E**). EGA increased the frequency of TNFα^+^ pDCs from some donors whereas it decreased the frequency of TNFα^+^ pDCs from others. Regardless of the frequency of intracellular TNFα^+^ pDCs, we found that EGA consistently decreased the amount of TNFα secreted from R837-stimulated (**Figure 1F**). In addition, EGA also decreased mRNA transcripts of both IFNα (**Figure 1G**) and TNFα (**Figure 1H**). It was of note that pDCs tested in **Figures 1F, H** were those that showed increased intracellular TNFα expression (**Figure 1E**) in the presence of EGA.

To further understand the TNFα expression data, showing discrepancy between the frequency of intracellular TNFα^+^ pDCs (**Figures 1D, E**) and the amount of TNFα in culture supernatants (**Figure 1F**) as well as the expression level of TNFα transcripts (**Figure 1H**), we assessed the frequency of surface pro-TNFα^+^ pDCs (**Supplementary Figure 1**). We found that the amount of TNFα secreted in the supernatants and TNF mRNA transcripts were consistent with the frequency of surface pro-TNFα^+^ pDCs, not intracellular TNFα^+^ pDCs (**Figures 1D, E**). It is known that newly made pro-TNFα is transported from the Golgi to the cell membrane where it can be cleaved by metalloproteinases to be released from cells (28, 29). However, it is also known that some of the pro-TNFα are endocytosed and traffic from early to late endosomes/lysosomes in which they can be degraded. As an endosomal trafficking inhibitor, EGA could inhibit such endocytosed pro-TNFα trafficking to late endosomes/lysosomes (30, 31), resulting in the accumulation of total TNFα intracellularly which could contribute to the increased frequency of intracellular TNFα^+^ pDCs. However, this needs to be further studied in the future. Similar to the R837-treated pDCs (**Supplementary Figure 1**), EGA also decreased surface pro-TNFα expression by pDCs stimulated with CpG DNAs (not shown). Anti-TNFα antibody used in this study can detect both pro-TNFα and active form of TNFα. We also found that EGA did not decrease the frequency of intracellular TNFα^+^ pDCs (**Supplementary Figure 2A**), the frequency of surface TNFα^+^ pDCs (**Supplementary Figure 2B**), or the amount of TNFα secreted by pDCs when pDCs were stimulated with R848. R848 is a TLR7/8 agonist and a high concentration of R848 (e.g., 2µg/ml used in this study) could mainly activate pDCs via TLR8, not TLR7 (32), but it remains to be better understood in the future.

We next tested whether EGA could also alter IL-6 expression by R837-stimulated pDCs, as both TNFα and IL-6 expression is largely dependent on the activation of NF-κB pathway upon TLR7 stimulation. We measured pDC expression of IL-6 by both intracellular staining (**Supplementary Figure 3A**) and assessing the amount of IL-6 in culture supernatants (**Supplementary Figure 3B**). First of all, we noticed that R837-stimulated pDCs expressed much lower levels of IL-6 than TNFα. In addition, EGA did not significantly affect such low levels of IL-6 expression by R837-stimulated pDCs. Although additional experiments need to be performed to explain our results in **Supplementary Figures 3A, B**, both secreted and intracellular TNFα might also affect IL-6 expression by pDCs incubated overnight. Similar to the IL-6 expression, EGA did not decrease CD86 expression level on R837-stimulated pDCs (**Supplementary Figure 3C**).

We also tested whether EGA could also decrease both IFNα and TNFα secretion by ssRNA40-stimulated pDCs. As shown in **Supplementary Figure 4A**, ssRNA40 complexed with DOTAP (33) induced IFNα secretion by pDCs and EGA decreased IFNα secretion. However, the amount of TNFα secreted by ssRNA40-stimulated pDCs was minimal (**Supplementary Figure 4B**), as reported in a previous study (33), showing that TNFα expression by ssRNA40 stimulation was mainly observed in CD11c^+^ cells in PBMCs of healthy blood samples.

Therefore, we conclude that EGA can modulate cytokine expression by R837- treated human blood pDC by decreasing the expression of IFNα and TNFα as well as the amount of TNFα secreted by pDCs stimulated with R837. EGA was also capable of decreasing IFNα secretion by pDCs stimulated with ssRNA40.

### 3.2 Inhibition of PIKfyve decreases IFNα expression by R837-stimulated pDCs

**Figure 2:**
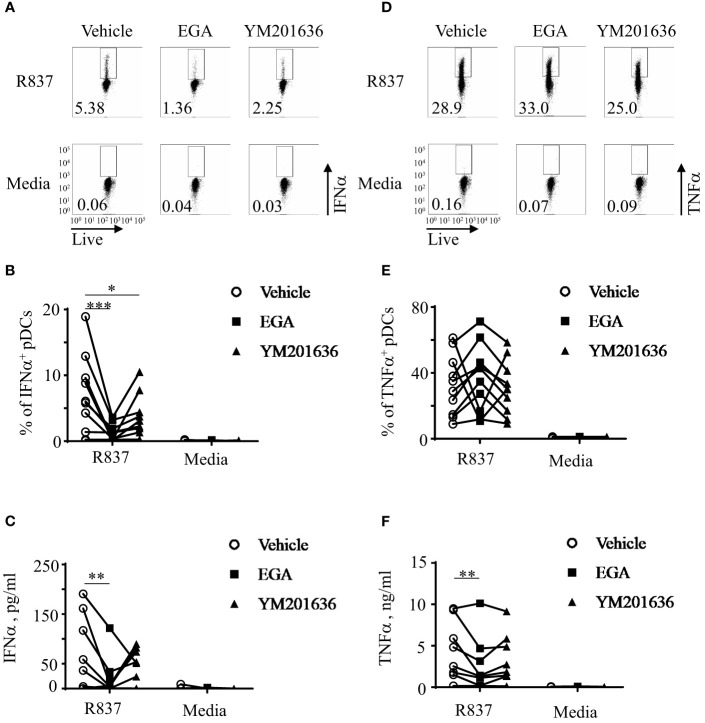
EGA is more effective than YM201636 at controlling IFNα expression by R837-stimulated pDCs. FACS-sorted pDCs were pre-incubated for 30 mins with 20 μM EGA, 1 μM YM201636, or vehicle. pDCs were then stimulated with 5 μg/mL R837 for 5 hours and stained for intracellular IFNα expression. Representative FACS plots **(A)** and summarized data of the frequency of IFNα^+^ pDCs **(B)**. After overnight culture, the amount of IFNα in the culture supernatants **(C)** was measured by bead-based cytokine assays. Representative FACS plots of the frequency of intracellular TNFα^+^ pDCs **(D)**, summarized data of the frequency of intracellular TNFα^+^ pDCs **(E)**, and the amount of TNFα secreted in the culture supernatants **(F)**. In **(B, C, E, F)**, individual lines indicate data generated with pDCs from different healthy subjects. Data analyzed by two-way ANOVA with Tukey multiple comparison test. *p <0.05, **p < 0.01, and ***p <0.001 for the comparison between groups.

Inhibition of PIKfyve activity can also result in the disruption of endo-lysosomal trafficking (34). It is also known that PIKfyve-mediated type 1 IFN expression by mouse macrophages can also induce ATF3 which negatively regulates type 1 IFN expression (35). Therefore, we tested whether the PIKfyve inhibitor, YM201636, would have similar effects as EGA on cytokine expression of R837-stimulated pDCs. Both EGA- and YM201636-treatments reduced IFNα expression in R837-stimulated pDCs; however, EGA was more effective than YM201636 (**Figures 2A, B**). As shown in **Figure 2C**, the amount of IFNα secreted by R837-stimulated pDCs was also decreased by EGA treatment.

In contrast to EGA, YM201636 did not significantly alter the frequency of intracellular TNFα^+^ pDCs (**Figures 2D, E**) or the amount of TNFα secreted in culture supernatants (**Figure 2F**) of R837-stimulated pDCs. Similar to **Figures 1E, F**, the effects of EGA treatment were variable among pDC donors when the frequency of intracellular TNFα^+^ pDCs was assessed. Unlike YM201636, EGA decreased the amount of TNFα secreted by pDCs when measured (**Figure 2F**) as in **Figure 1F**. Altered cytokine expression by EGA- and YM201636-treatments were not due to the effects on pDC viability (**Supplementary Figure 5**).

We concluded that both EGA and YM201636 alters IFNα expression by pDCs stimulated via TLR7, but EGA was more effective than YM201636 at reducing IFNα expression by R837-stimulated pDCs. EGA was also more effective than YM201636 at decreasing the amount of TNFα secreted by R837-stimulated pDCs.

### 3.3 EGA suppresses IKKα/β, p38, and STAT1 phosphorylation, but prolongs IκBα degradation in R837-stimulated pDCs

To investigate the mechanisms of action of EGA as well as differences with YM201636 at altering R837-stmulated pDCs, we assessed kinases and regulatory proteins that are known to be involved in cytokine expression by pDCs. These kinases included IKKα/β activation (36, 37) for type 1 IFN expression, IκBα (38) and p65 (39) for pro-inflammatory cytokine expression, p38 and STAT1 phosphorylation for interferon-stimulated gene factor 3 (ISGF3) formation (40, 41) which controls IFN-inducible gene expression, and IRF7 as a regulator of type 1 IFN expression (42). We also assessed whether ATF3 was enhanced with EGA- and YM201636-treatments as a possible mechanism for depressed type 1 IFN expression (35, 43). ATF3 can modulate IFN response as well as the expression of genes downstream of IFN signaling (35, 43). In addition, blocking the trafficking process through PIKfyve inhibition could rapidly induce the expression of the transcription repressor ATF3, resulting in the suppression of type I IFN expression by pDCs (35, 43).

**Figure 3:**
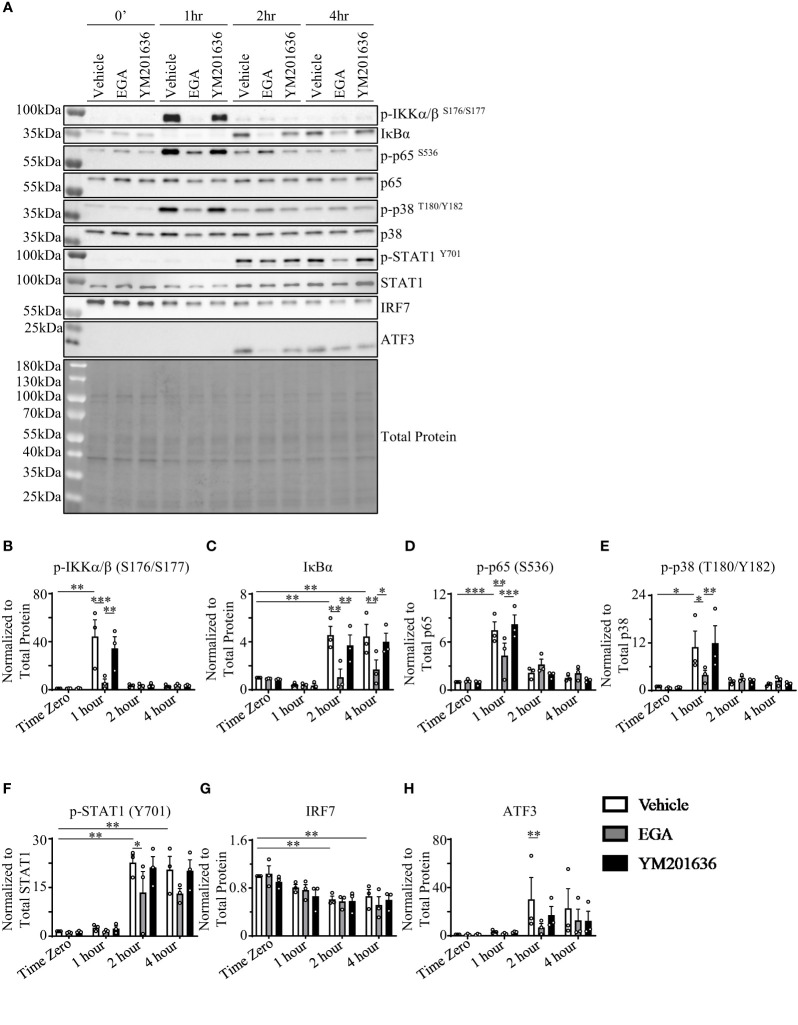
EGA inhibits activation of IKKα/β, p38, and STAT1 but prolongs IκBα degradation. FACS-sorted pDCs were rested for 1 hour before treating them with 20 μM EGA, 1μM YM201636, or vehicle for 30 mins. Cells were then stimulated with 5 μg/mL R837 for the indicated time. Representative blot **(A)** with quantified changes in p-IKKα/β **(B)**, IκBα **(C)**, p-p65 **(D)**, p-p38 **(E)**, p-STAT1 **(F)**, IRF7 **(G)**, and ATF3 **(H)** from 3 independent experiments utilizing pDCs isolated from different donors. Inhibitor-mediated changes in protein data analyzed by two-way ANOVA with Tukey multiple comparison test. *p < 0.05, **p < 0.01, and ***p < 0.001 are for the comparison between groups.

Similar to the previously reported data generated with DCs, both mDCs and pDCs (36), and pDC cell line, Gen2.2 (37), R837-stimulation increased IKKα/β phosphorylation in pDCs. However, this increase was transient (**Figures 3A, B**). As shown in **Figure 3C**, IκBα levels were too low to examine the effects of EGA treatment by 1 hr. The effects of EGA on IκBα levels were seen at 2 hrs after activation. In contrast, IKKα/β activation and EGA-mediated suppression of IKKα/β activation was observed at 1 hr (**Figure 3B**). Interestingly, EGA treatment significantly reduced R837-induced IKKα/β phosphorylation while YM201636 treatment did not. This EGA-induced suppression of IKKα/β activation followed by reduced secretion of IFNα from R837-stimulated pDCs is supported by the data from previous studies (36, 37), showing that both IKKα and IKKβ contribute to type 1 IFN expression by pDCs. Notably, EGA-treatment also decreased IκBα accumulation after degradation (**Figures 3A, C**). Our results showed that EGA suppresses IKKα/β phosphorylation but prolongs IκBα degradation in R837-stimulated pDCs. This data could be surprising as degradation of IκBα could be the result of IKKα/β phosphorylation. However, previous studies have shown that IkBα degradation could also be independent of IKKα/β phosphorylation (44, 45). p65 (**Figures 3A, D**) and p38 (**Figures 3A, E**) phosphorylation also occurred at 1 hour in vehicle- and YM201636-treated pDCs with EGA-treatment suppressing phosphorylation of both p65 and p38 at 1 hour. These differences were not observed after 1 hour. STAT1 phosphorylation (**Figures 3A, F**) was enhanced 2 hours post-R837-stimulation with EGA-treatment inhibiting its phosphorylation. R837-stimulation of pDCs caused a decline in IRF7 levels at 2 and 4 hours; however, this decline occurred in all groups (**Figures 3A, G**). ATF3 expression was upregulated after IKKα/β phosphorylation and had similar kinetics to STAT1 phosphorylation in all treated groups (**Figure 3A**), suggesting autocrine effects of type 1 IFN expression (43) instead of an effect of EGA- or YM201636-treatments (35, 43). However, EGA-treatment inhibited ATF3 (35, 43) upregulation compared to vehicle (**Figure 3H**) further confirming EGA diminished the expression of type 1 IFN.

Taken these data together, we concluded that EGA could affect multiple kinases involved in R837-induced cytokine responses by pDCs. EGA-treatment suppressed the phosphorylation of IKKα/β, p38, and STAT1 which are involved in the expression of type 1 IFN. This was further confirmed through EGA-mediated reduction of ATF3 upregulation, which is an interferon-inducible gene (43). EGA-treatment suppressed p65 phosphorylation at early time point (1 hour). EGA also suppressed IκBα, supporting the decreased secretion of TNFα by R837-activated pDCs treated with EGA.

### 3.4 EGA blocks the trafficking of TLR7 ligand, 3F, to LAMP1^+^ endo-lysosomes

We next investigated whether EGA could alter the trafficking of TLR7 agonists in pDCs which could affect cytokine response by pDCs. We employed a previously characterized TLR7-specific substituted adenine, 3F (23, 46) that has a similar TLR7 activity to the imidazoquinoline (R848) and R837 (23, 46).

**Figure 4:**
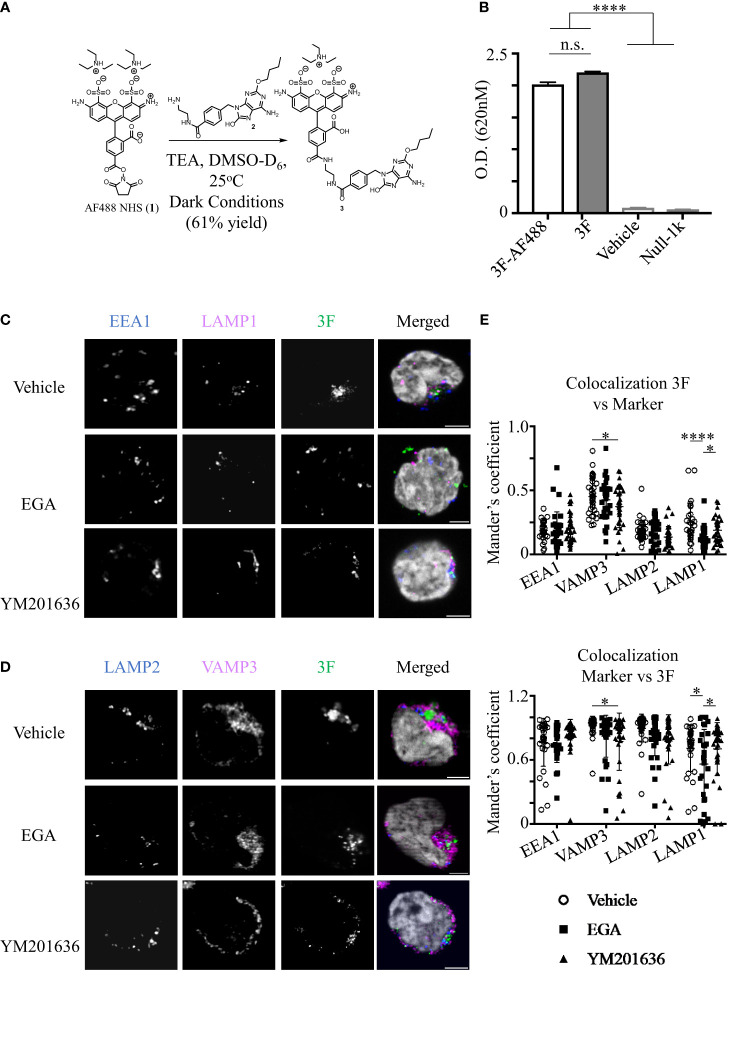
EGA inhibits 3F-AF488 colocalization with LAMP1^+^ compartments in pDCs. **(A)** Reaction conditions for conjugation of 3F compound to Alexa Fluor 488. **(B)** HEK Blue hTLR7-expressing cells were stimulated with 10 μM 3F and 3F-AF488, and vehicle for 15 hours. SEAP expression was measured by optical density to assess NF-κB activity. Parental cell line (Null-1k) of hTLR7-expressing cells did not express secreted embryonic alkaline phosphatase (SEAP) in response to 3F stimulation. Representative data from experiment performed in triplicate assay. **(C–E)** pDCs were pre-incubated with 20 μM EGA, 1μM YM201636 or vehicle for 1 hour before addition of 20 μM 3F-AF488 for 5 hours. **(C, D)** Representative staining of endo-lysosomal markers and 3F-AF488 within pDCs. **(E)** Summarized colocalization data of 3F-AF488 with each endo-lysosomal marker in 30-34 different cells from 3 different experiments. Scale bar - 2μm. Data analyzed by one way ANOVA **(B)** or two-way ANOVA **(E)** with Tukey’s multiple comparison test. *p < 0.05, ****p <0.0001, and n.s., not significant for comparison between groups.

3F was conjugated with AF488 as depicted in **Figure 4A**. 3F-AF488 was purified by a high-pressure liquid chromatography (HPLC) (**Supplementary Figure 6**). Purified 3F-AF488 was further characterized with a nuclear magnetic resonance (NMR) spectroscopy (**Supplementary Figure 7**). There was no free TLR7 ligand.

Reporter cell assay using HEK-Blue TLR7 cells showed that both 3F and 3F-AF488 conjugates were comparable at activating HEK-Blue cells via the ligation of TLR7 (**Figure 4B**), as there was no activation of parental cell line (Null-1k) treated with 3F.

We next investigated whether EGA would alter trafficking of 3F-AF488 in pDCs by examining its colocalization with EEA1, VAMP3, LAMP2 and LAMP1. After analyzing the kinetics of 3F-AF488 uptakes (**Supplementary Figures 8A**), we performed experiments at 5 hours after 3F-AF488 treatment. We found that 3F-AF488 had reduced colocalization with LAMP1^+^ compartments in EGA-treated cells when compared to either vehicle- or YM201636-treatments (**Figures 4C–E**). EGA did not affect the colocalization of 3F-AF488 with other endo-lysosomal markers. YM201636-treatment did affect the colocalization of 3F-AF488 with VAMP3^+^ compartments compared to vehicle-treatment (**Figure 4E**) although this was not significant when compared to EGA-treatment (**Figure 4E**). EGA did not reduce the accessibility of 3F-AF488 into pDCs (**Supplementary Figures 8B, C**).

Therefore, we concluded that EGA-treatment could inhibit the trafficking of the small TLR7 agonists to LAMP1^+^ compartments. As previously described (17), TLR7 accumulation in late endosomes and lysosomes was required for type 1 IFN expression by pDCs stimulated with TLR7 agonists. The inhibition of 3F trafficking to LAMP1^+^ compartments by EGA (**Figure 4**) support the EGA-mediated decreased IFNα expression by R837-stimulated pDCs (**Figures 1**, **2**). Another study also reported that TLR7 localization was increased in late endosomes/lysosomes in pDCs of SLE patients (47). The enhanced IFNα production by TLR7-stimulated SLE pDCs was associated with increased retention of TLR7 in late endosomes/lysosomes in SLE pDCs (47). EGA treatment did not alter 3F colocalization with other endo/lysosomal markers, including EEA1, LAMP2, and VAMP3.

### 3.5 EGA inhibits IFNα expression by R837-stimulated SLE pDCs

Previous studies (47, 48) reported that pDCs from SLE patients, when compared to pDCs of control subjects, have an enhanced cytokine response to TLR7 agonists. Such altered phenotype of SLE pDCs correlated with an increased colocalization of TLR7 within late endosomes (47). Therefore, we investigated how EGA affects TLR7-mediated cytokine responses by pDCs from the blood of SLE patients. Aberrant activation of TLR7 has also been associated with the interferon signatures in SLE (49–52) and with the pathogenesis of SLE (13, 47, 53).

**Figure 5:**
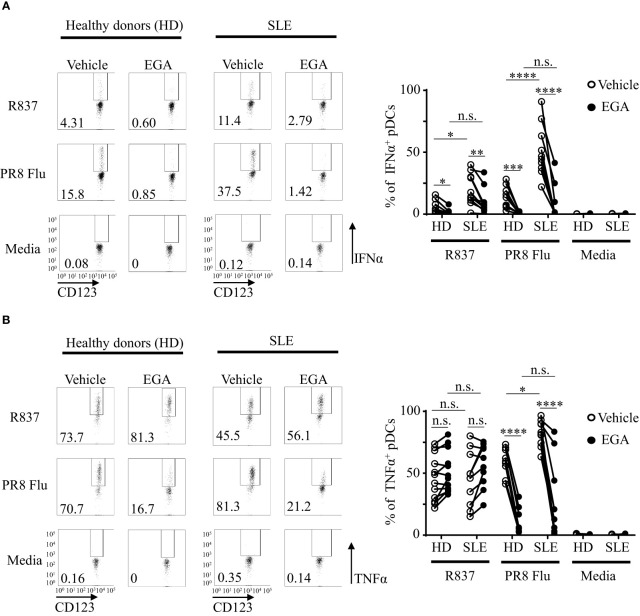
EGA reduces IFNα expression by SLE pDCs stimulated with R837 and PR8 influenza virus. PBMCs from SLE patients and healthy subjects were pre-incubated for 30 mins with 20 μM EGA or DMSO vehicle. Cells were then stimulated with 5 μg/mL R837 or 2 MOI PR8 influenza virus (A/PR8/34, H1N1) for 5 hours before staining them for intracellular IFNα and TNFα expression. Representative FACS plots for IFNα (**A,** left panels) and TNFα (**B,** left panels) expression by pDCs of healthy donors (HD) and SLE patients. Summarized data for IFNα (**A,** right panel) and TNFα (**B,** right panel). Individual lines indicate data generated with PBMCs from different subjects. Data analyzed by 2-way ANOVA with Tukey multiple comparison test. *p < 0.05, **p < 0.01, ***p < 0.001, ****p <0.0001, and n.s., not significant for comparison between groups.

With limited volume of blood samples from SLE patients, experiments in this section were performed with PBMCs. After *in vitro* cultures of PBMCs as indicated, pDCs were gated as in **Supplementary Figure 9A**. **Figure 5A** shows that EGA-treatment reduced IFNα expression by pDCs from both healthy and SLE patients in response to both R837 and PR8 influenza virus. Summarized data (right panel, **Figure 5A**) also show that SLE pDCs were more potent than healthy pDCs at expressing IFNα in response to both R837 and PR8 influenza viruses, and this is in line with the previously published data (47, 48), showing that greater numbers of SLE pDCs expressed IFNα than pDCs from healthy controls in response to TLR7 ligands. Similar to the data generated with purified pDCs (**Figures 1**, **2**), EGA treatment did not significantly reduce or increase the frequency of intracellular TNFα^+^ pDCs when they were stimulated with R837 (right panel, **Figure 5B**). The effects of EGA were also variable among donors. However, EGA reduced the frequency of intracellular TNFα^+^ pDCs when they were stimulated with PR8 influenza viruses. As previously described (22), EGA inhibits influenza viral uncoating by blocking their trafficking to late endosomes and lysosomes.

**Figure 6:**
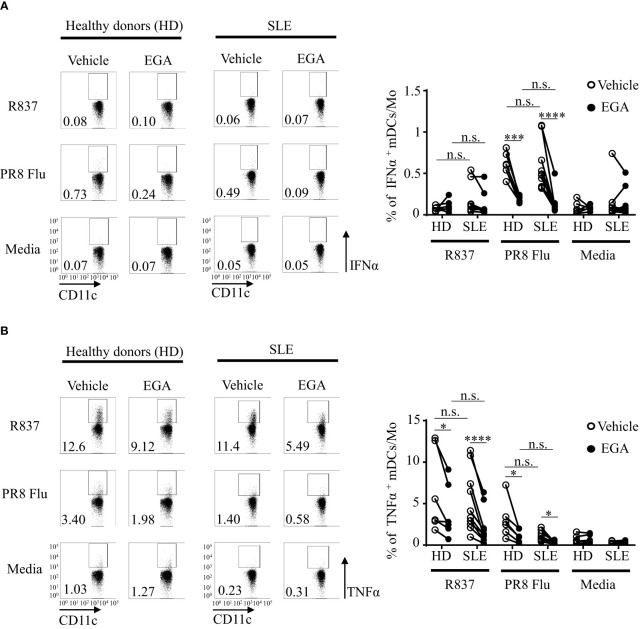
EGA controls PR8 influenza virus-induced IFNα and R837-induced TNFα expression by mDC/monocyte population isolated from SLE patients. PBMCs from SLE patients and healthy subjects were pre-incubated for 30 mins with 20 μM EGA or vehicle. Cells were then stimulated with 5 μg/mL R837 or 2 MOI PR8 influenza virus (A/PR8/34, H1N1) for 5 hours before staining them for intracellular IFNα and TNFα expression. Representative FACS data for IFNα (**A**, left panels) and TNFα (**B**, left panels) expression by mDC/monocyte population from healthy donors (HD) and SLE patients. Summarized data for IFNα (**A**, right panel) and TNFα (**B**, right panel). Individual lines indicate data generated with PBMCs from different subjects. Data analyzed by 2-way ANOVA with Tukey multiple comparison test *p < 0.05, ***p < 0.001, and ****p <0.0001 and n.s., not significant for comparison between groups.

We further analyzed cytokine expression by mDCs/monocyte populations (CD3^-^CD19^-^CD56^-^HLA-DR^+^CD11c^+^CD123^-^ in **Supplementary Figures 9A, B**). As shown in **Figure 6A**, the frequency of intracellular IFNα^+^ mDCs/monocytes were minimal (less than 1%) when they were stimulated with R837, and there was no significant effect of EGA treatment (right panel in **Figure 6A**). However, PR8 influenza virus-induced IFNα expressions by mDCs/monocytes from both healthy individuals and SLE patients were decreased by EGA treatment.

Although the frequency of TNFα^+^ mDCs/monocytes (less than 15%, **Figure 6B**) are less than the frequency of TNFα^+^ pDCs (25-70%, **Figure 5B**), EGA could decrease the frequency of intracellular TNFα^+^ mDCs/monocytes stimulated with R837 and PR8 influenza virus. mDCs/monocytes from healthy subjects and SLE patients showed similar results. It was of note that the effects of EGA treatment on the frequency of intracellular TNFα^+^ pDCs were variable among donors (**Figures 1E**, **2E**), but EGA could consistently decrease the frequency of intracellular TNFα^+^ mDCs/monocytes (**Figure 6B**), suggesting that cellular machineries for TLR7-mediated cytokine expression and/or secretion might not be the same in all cell types that will need to be further investigated in future studies. For example, in addition to the potential differences in the types and activation of membrane proteinases, cell type-specific metabolomic machineries are also known to differentially control inflammatory mediators, including TNFα, in human DC subsets, pDCs versus mDCs (54).

We therefore concluded, EGA can suppress IFNα expression by SLE pDCs treated with R837 and influenza virus. EGA can also effectively suppress PR8 influenza virus-induced TNFα expression. EGA is also capable of suppressing PR8 influenza virus-induced IFNα as well as R837-induced TNFα expression by mDCs/monocytes.

## 4 Discussion

pDCs can exhibit a bifurcated cytokine response to TLR9 agonists based on the ability of agonists to form higher order structures which coincides with their localization within distinct signaling endosomes in pDCs (18, 20, 55). However, how the small molecule TLR7 agonists, imidazoquinolines and substituted base analogs, traffic in pDCs remains to be better understood. Furthermore, regulation of the cytokine expression induced by TLR7 agonists may be beneficial in reducing the exacerbation of certain types of autoimmune diseases (56–59), including SLE. The cellular entry of imidazoquinolines and substituted adenines have been shown to take part in two steps: a passive diffusion entry step and then an active step involving the vacuolar-ATPase enhancing the accumulation within a LAMP1^+^ compartments co-expressing CD63 and MHCII (17). However, these experiments were performed on pDCs rested overnight in IL-3 (17) which do not exhibit bifurcated cytokine responses (17, 60). Therefore, data generated in this study extend our knowledge on how pDCs respond to small molecule TLR7 agonists, and further support additional studies testing the effectiveness of EGA, an endosomal trafficking inhibitor, in the regulation of TLR7-mediated cytokine response in the near future.

EGA, a late endosome trafficking inhibitor, decreased IFNα expression and secretion by pDCs stimulated via TLR7. This was demonstrated by measuring IFNα transcript, intracellular IFNα expression, and the amount of IFNα secreted in the culture supernatants. EGA also decreased PR8-influenza virus-induced IFNα expression by inhibiting their trafficking to late endosome/lysosome, resulting in the inhibition of viral uncoating, as previously described (22). Similar to IFNα, EGA also decreased the expression of TNFα transcripts as well as the amounts of TNFα secreted in the culture supernatants of R837-stimulated pDCs. Interestingly, however, this was not the case when we assessed the frequency of intracellular TNFα^+^ pDCs. We further found that EGA could decrease the frequency of pDCs expressing pro-TNFα on the cell membrane (28, 29), which was in line with the expression level of TNFα transcripts and the amount of TNFα secreted. Pro-TNFα are released from membrane by the action of metalloproteinases (28, 29), but some of them are endocytosed and traffic to late endosomes/lysosomes where they are degraded (30, 31). Therefore, the increased frequency of intracellular TNFα^+^ pDCs could be due to the inhibitory action of EGA on the trafficking of pro-TNFα to late endosomes/lysosomes followed by reduced degradation of pro-TNFα. Anti-TNFα used in this study bind to both pro-TNFα and active form of TNFα. However, this needs to be further studied in the future. It is also of note that EGA could consistently reduce the frequency of intracellular TNFα^+^ pDCs, which was in line with the amount of TNFα secreted in culture supernatants, when they were stimulated with CpG-ODNs (27). Therefore, additional studies, including the expression and activation of different proteinases that could also be affected by different stimuli, e.g., TLR ligands, as well as the effects of autocrine cytokines, e.g., IFNα and TNFα (61), on proteinase activities (62), need to be performed in the future. In addition, different cell types might have shared as well as distinct cellular and molecular mechanisms for the secretion of an active form of TNFα, as EGA did not significantly affect the frequency of intracellular TNFα^+^ pDCs (**Figure 5B**) while effectively decreasing intracellular TNFα^+^ monocytes/mDCs (**Figure 6B**). It is also known that TLR7 expression levels could be variable depending on seasons (63), e.g., summer versus winter, sexes, and other factors including cigarette smoke (64) and estrogen receptor signaling (65).

While both EGA (22) and the PIKfyve inhibitor, YM201636 (34), have been known to block trafficking to lysosomal compartments, our data indicated that EGA was more effective than YM201636 at inhibiting IFNα expression by R837-stimulated pDCs. Despite EGA treatment ability to suppress IFNα expression, EGA did not enhance ATF3 expression ruling out this as a possible mechanism for type 1 IFN suppression. In support of the minimal effect of YM201636 treatment on IFNα expression, YM201636 did not significantly alter ATF3 expression or activation of p38, IKKα/β, and STAT1.

As previously documented (17), fluorophore-conjugated imidazoquinoline altered imidazoquinoline TLR7 activity; however, fluorophore conjugates of TLR7 agonists, substituted adenines, did not exhibit significantly altered TLR7 activity. We thus employed 3F-AF488 to investigate the effects of EGA on the trafficking of TLR7 ligands. 3F-AF488 conjugates showed comparable levels of TLR7 activity induced by 3F. We found that only EGA, but not YM201636, was effective at reducing colocalization of 3F-AF488 with LAMP1^+^ compartments in pDCs. This conforms with previous report (17) showing that small molecule TLR7 agonists accumulate and signal within this acidic compartment due to the activity of the v-ATPase. In addition to IFNα and TNFα, we also measured the amount of IL-1β secreted by R837-stimulated pDCs. We found that R837-stimulated pDCs did not secrete significant amount (less than 5-10 pg/ml from 2x10^5^ pDCs) of IL-1β. EGA or YM201636 did not alter IL-1β expression by R837-stimulated pDCs (not shown). IL-1β expression is known to be increased in endoplasmic reticulum (ER) stress (66) This suggested that EGA treatment did not induce ER stress or inflammasome activation. A previous study (67) reported an imiquimod-induced ER stress, but this phenomenon was imiquimod concentration dependent. ER stress was induced with high concentration (10-100 μg/ml) of imiquimod only. 5μg/mL of imiquimod was used in this study.

EGA treatment resulted in a greater reduction of IFNα expression by pDCs treated with influenza virus, compared to pDCs treated with R837. In addition, EGA inhibited both IFNα and TNFα expression by influenza virus-treated pDCs, which is in contrast to R837-treated pDCs. This could be explained by that, after endocytosis, influenza virus requires a low pH-dependent fusion to release viral genomes. Indeed, EGA can inhibit entry of virus particles to the cellular compart with a low pH (22).

An increased TLR7 localization in Rab7^+^ and LAMP1^+^ compartments in SLE pDCs was previously reported (47). As EGA blocked the trafficking of substituted adenines to LAMP1^+^ compartments, we assessed the efficacy of EGA treatment in altering TLR7 responses in SLE pDCs. Similar to previous studies (47, 48), SLE pDCs were more effective than pDCs from healthy subjects at expressing IFNα in response to TLR agonists (**Figure 5A**). However, EGA treatment was still capable of suppressing IFNα and TNFα expression by SLE pDCs treated with R837 and PR8 influenza virus. As it has been proposed that TNFα and IFNα signaling pathways cross-regulate each other (68–71), EGA treatment might be beneficial by suppressing both IFNα and TNFα in autoimmunity, although this remains to be adequately determined (71). It was also of note that EGA was capable of suppressing PR8 influenza virus-induced IFNα expression as well as R837-induced TNFα expression by mDC/monocyte population from both SLE patients and healthy subjects. Although this study focused on the effects of EGA on the SLE pDC cytokine response to TLR7 ligand only, additional studies, e.g., testing the potential effects of EGA on immune complex-mediated cytokine expression and SLE pathogenesis, are warranted in the future. It is also important to note that EGA, an endosomal trafficking inhibitor, targets not only pDCs, but also other cell types. Potential advantages of using EGA might thus include that it can suppress type 1 IFN expression by pDCs as well as other inflammatory cytokine expression by other cell types, including monocytes and mDCs that also express TLR7. EGA does not affect pDC viability, which could be another potential advantage over pDC depletion strategies. However, EGA could also lead to the impairment of host immunity against many viral infections by dampening type 1 IFN responses (9, 10, 72).

In summary, EGA-treatment alters cytokine expression by pDCs stimulated with small TLR7 agonists as well as TLR9 agonists, including CpG-ODNs and genomic DNAs (27).This effect is likely due to EGA treatment affecting the trafficking of these compounds to LAMP1^+^ (for TLR7 ligands) or LAMP1^+^/LAMP2^+^ (for TLR9 ligands) subcellular compartments in pDCs. EGA may therefore serve as another potent immune modulator and might also have potential efficacy in controlling cytokine responses of pDCs in SLE, as we demonstrated that EGA can diminish IFNα expression by pDCs stimulated with TLR7 and TLR9 (27) ligands. Additional studies examining the potential benefits of EGA in treating type 1 IFN-associated inflammatory diseases are warranted in the future.

## Data availability statement

The original contributions presented in the study are included in the article/**Supplementary Material**. Further inquiries can be directed to the corresponding author.

## Ethics statement

The studies involving humans were approved by Mayo Clinic Institutional Review Board. The studies were conducted in accordance with the local legislation and institutional requirements. Patient samples used in this study were acquired with written informed consents in accordance with the national legislation and institutional requirements.

## Author contributions

MW and LB designed and carried out experiments. KG and RK synthesized and characterized the 3F compound and the 3F-AF488 conjugate. MK, LWG, and, HJ identified SLE patients. MK recruited patients and provided the SLE patient samples for this study. MW, LB, CG, HJ, LWG, J-PG, DB, MK, LWG, HJ, and SO analyzed the data. MW, LB, and SO wrote the manuscript. All authors contributed to the article and approved the submitted version.
