# Supplementary material for: Endosomal trafficking inhibitor EGA can control TLR7-mediated IFNα expression by human plasmacytoid dendritic cells
Source: Front Immunol. 2023 Nov 24;14:1202197. doi: 10.3389/fimmu.2023.1202197 (PMC10704457; doi:10.3389/fimmu.2023.1202197)
Supplement: Supplementary file 1 [file DataSheet_1.pdf]

frontiers

Supplementary Material

Endosomal trafficking inhibitor EGA can control TLR7-mediated IFNα expression by human plasmacytoid dendritic cells

Matthew J Wiest[1]*, Laurie Baert[1]*, Chao Gu[1], Kevin M Gayler[2], Hyoungjun Ham[3], Laurent Gorvel[4], Mira T Keddis[5], Leroy W Griffing[6], HyeMee Joo[1,2], Jean-Pierre Gorvel[7], Daniel D Billadeau[3], Robert R Kane[2], SangKon Oh[1, 2*]

[1] Department of Immunology, Mayo Clinic, Scottsdale, AZ, USA

[2] Department of Chemistry and Biochemistry, Baylor University, Waco, TX, USA

[3]Department of Immunology, Mayo Clinic, Rochester, MN, USA

[4]CRCM, Aix Marseille Universite, INSERM, Marseille, France

[5]Department of Nephrology, Mayo Clinic, Scottsdale, AZ, USA

[6]Department of Rheumatology, Mayo Clinic, Scottsdale, AZ, USA

[7]Aix Marseille Univ, CNRS, INSERM, CIML, Marseille, France

* MW and LB equally contribute to this manuscript.

Correspondence: SangKon Oh, Ph.D: Oh.Sangkon@mayo.edu

This file includes:

Supplementary Figure 1. Surface TNFα expression by R837-stimulated pDCs.
Supplementary Figure 2. EGA does not affect TNFα expression or secretion by R848-stimulated pDCs.
Supplementary Figure 3. EGA does not affect IL-6 and CD86 expression by R837-stimulated pDCs.
Supplementary Figure 4. EGA decreases IFNα secretion by ssRNA40-stimulated pDCs.
Supplementary Figure 5. EGA and PIKfyve inhibitor, YM201636, do not affect pDC viability.
Supplementary Figure 6. HPLC analysis of 3F-AF488 conjugate (in Figure 4A).
Supplementary Figure 7. NMR spectra of 3F-AF488 conjugate (compound 3 in Figure 4A).
Supplementary Figure 8. Kinetics of 3F-AF488 conjugate uptakes by pDCs.
Supplementary Figure 9. Gating strategy for analysis of intracellular cytokine expression in pDCs and mDCs/monocytes.
Supplementary Table 1. Antibodies utilized in flow cytometry in this study.
Supplementary Table 2. Antibodies utilized in immunoblotting in this study.
Supplementary Table 3. Antibodies utilized in confocal microscopy in this study.
Supplementary Table 4. Information of SLE patients recruited in this study.

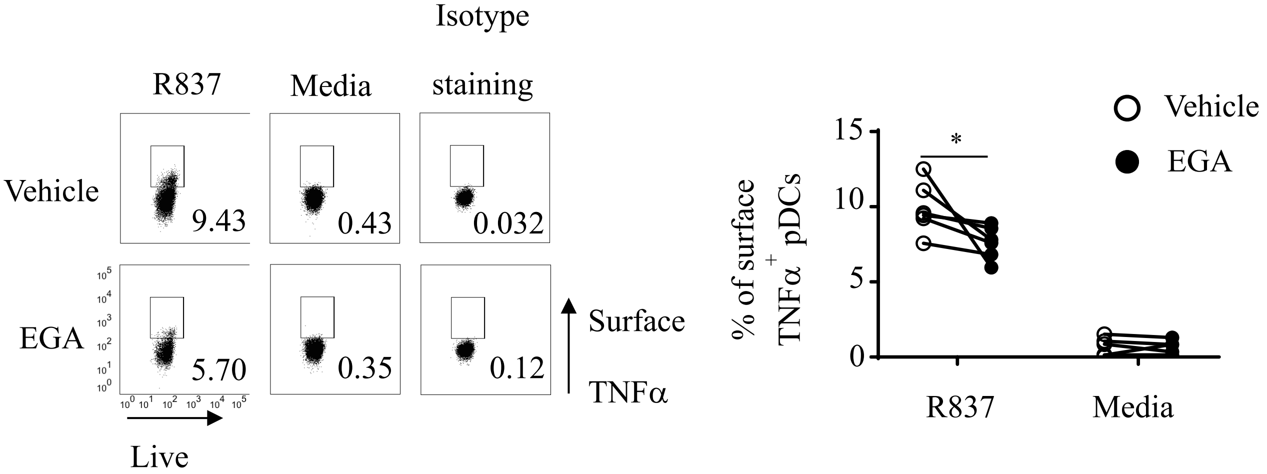

Supplementary Figure 1. Surface TNFα expression by R837-stimulated pDCs.

Purified pDCs were pre-incubated with 20 μM EGA or vehicle, and then stimulated with R837 for 5 hours. pDCs were stained for surface TNFα expression. Live cells were gated and analyzed. Left panels: Representative flow cytometry plots from three experiments testing pDCs from 6 healthy donors. Right panel: Summarized data. Each line represents data generated with pDCs of one donor. Data analyzed by paired t-test. *$p < 0.05$.

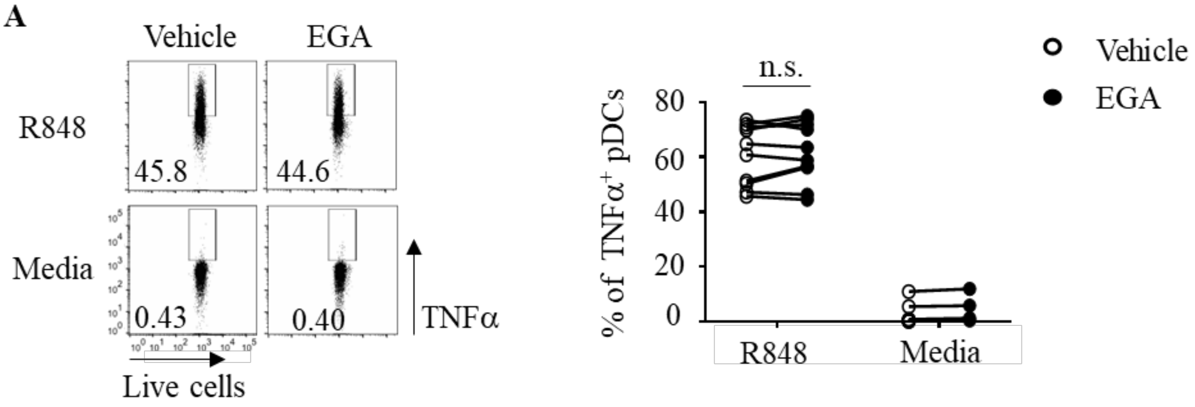

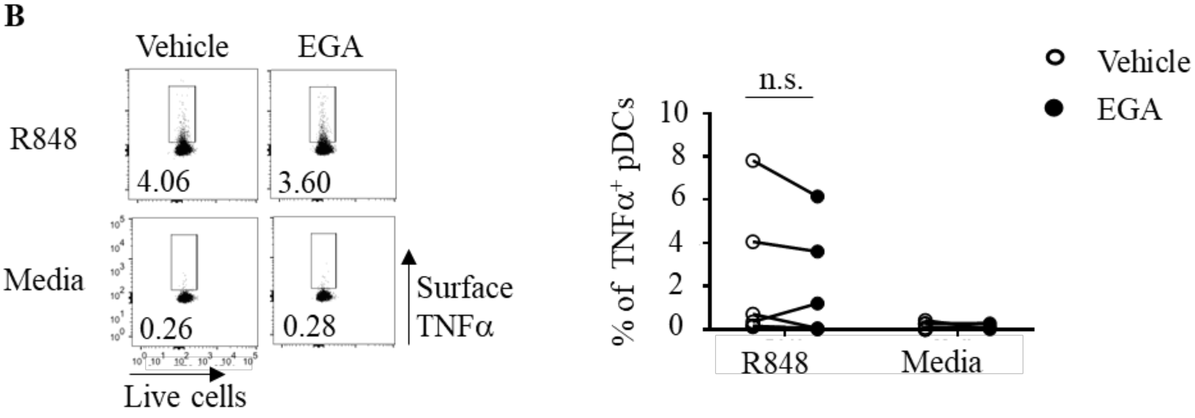

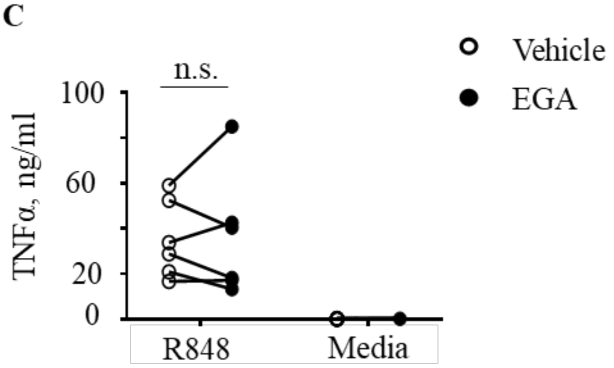

Supplementary Figure 2. EGA does not significantly affect TNFα expression or secretion by R848-stimulated pDCs.

Purified pDCs were pre-incubated with 20 µM EGA or vehicle, and then stimulated with 2 µg/ml R848 for 5 hours. Intracellular TNFα (A) and surface TNFα (B) expression was assessed. Representative flow cytometry plots (left panel) and summarized data generated with pDCs of 6-10 healthy donors (right panel) are presented. (C) After overnight culture, the amount of TNFα in culture supernatant was analyzed. pDCs isolated from 6 healthy subsets were tested. Data analyzed by paired t-test. n.s.: non-significant.

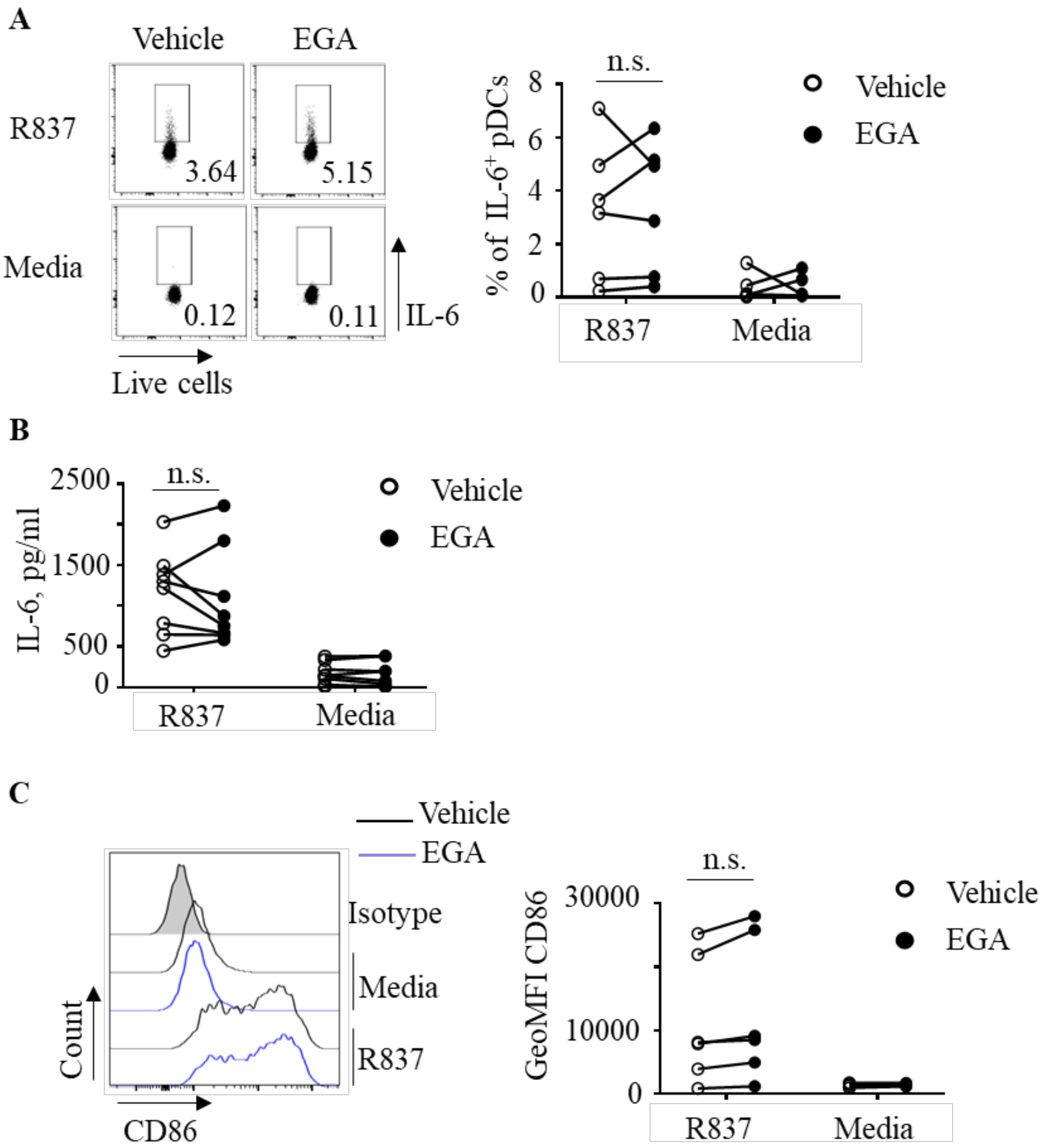

Supplementary Figure 3. EGA does not affect IL-6 and CD86 expression by R837-stimulated pDCs.

Purified pDCs were pre-incubated with 20 μM EGA or vehicle, and then stimulated with R837 for 5 hours. (A) Intracellular IL-6 expression was assessed. Representative flow cytometry plots (left panel) and summarized data generated with pDCs of 6 healthy donors (right panel) are presented. (B) After overnight culture, the amount of IL-6 in culture supernatant was assessed. (C) After overnight culture, surface CD86 expression levels were measured. Representative flow cytometry plots (left panel) and summarized data generated with pDCs of 6 healthy donors (right panel) are presented. Data analyzed by paired t-test. n.s.: non-significant.

A

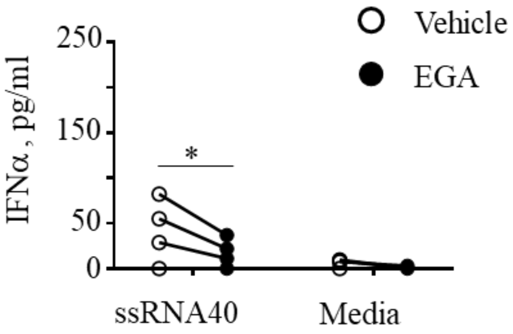

B

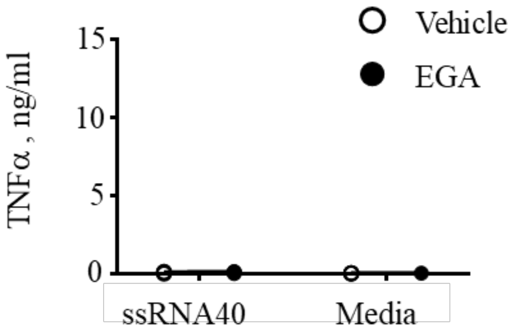

Supplementary Figure 4. EGA decreases IFNα secretion by ssRNA40-stimulated pDCs.

Sorted pDCs were pre-incubated with 20 µM EGA, or vehicle, and then stimulated with 2 µg/mL ssRNA40 previously conjugated with 10 µg/ml DOTAP for 5 hours. After overnight culture, the amount of IFNα (A) and TNFα (B) in culture supernatant was assessed. Data analyzed by paired t-test. *$p < 0.05$.

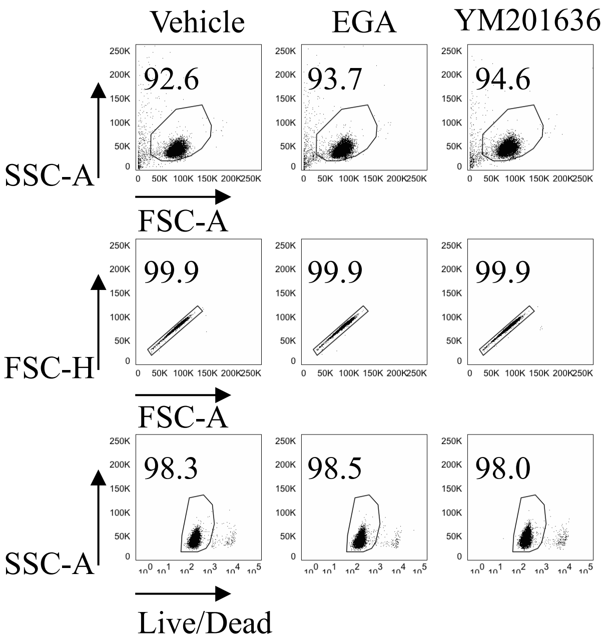

Supplementary Figure 5. EGA and PIKfyve inhibitor, YM201636, do not affect pDC viability.

pDCs pre-incubated with 20 μM EGA, 1 μM YM201636, or vehicle, stimulated with R837 for 5 hours and stained for viability. Representative flow cytometry plots from five experiments.

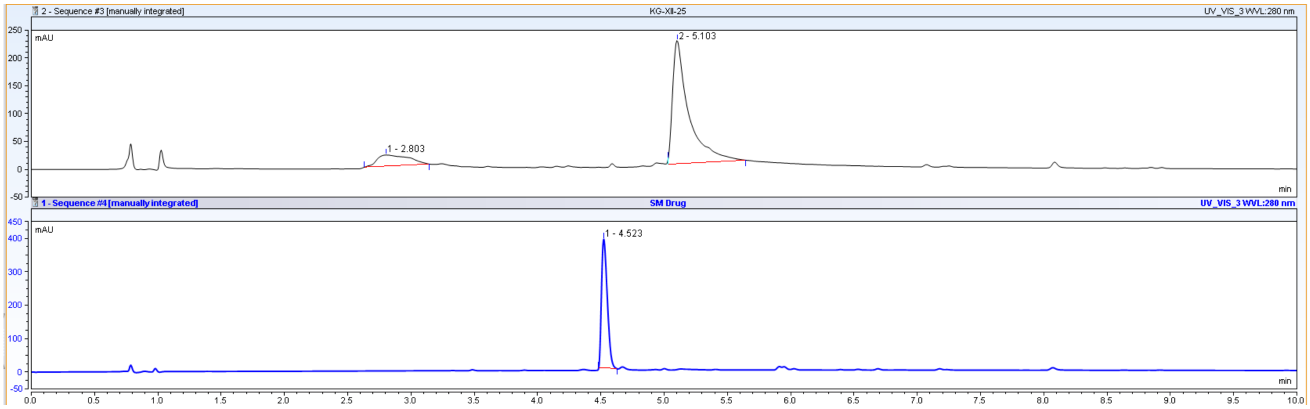

Supplementary Figure 6. HPLC analysis of 3F-AF488 conjugate (in Figure 4A).

280 nm trace. Top: 3F-AF488 conjugate (compound 3 in Figure 4A). Bottom: 3F alone

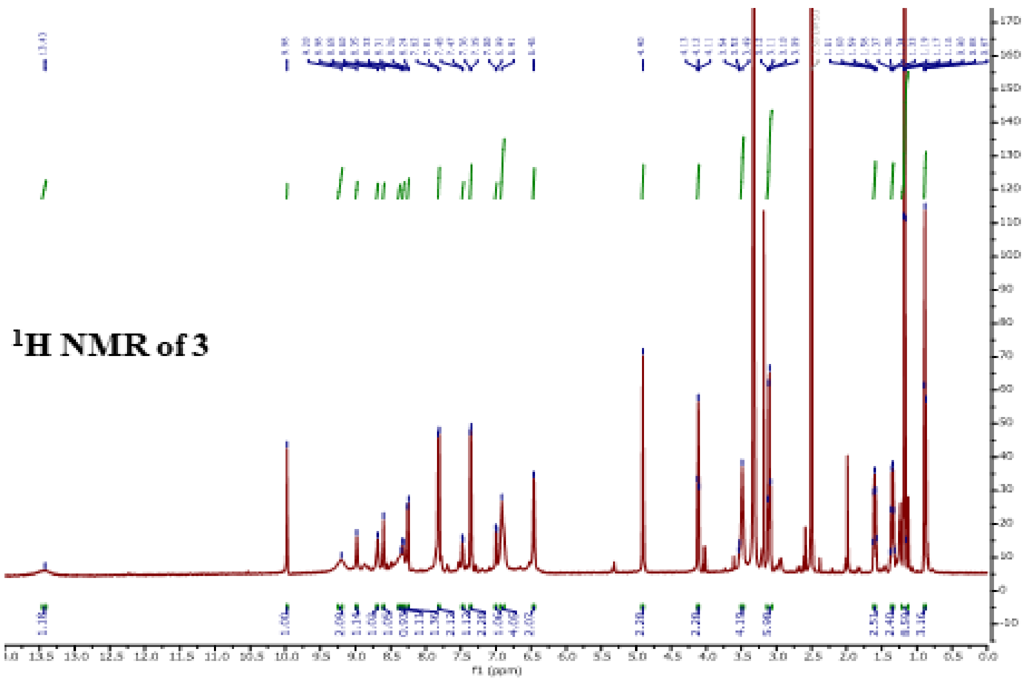

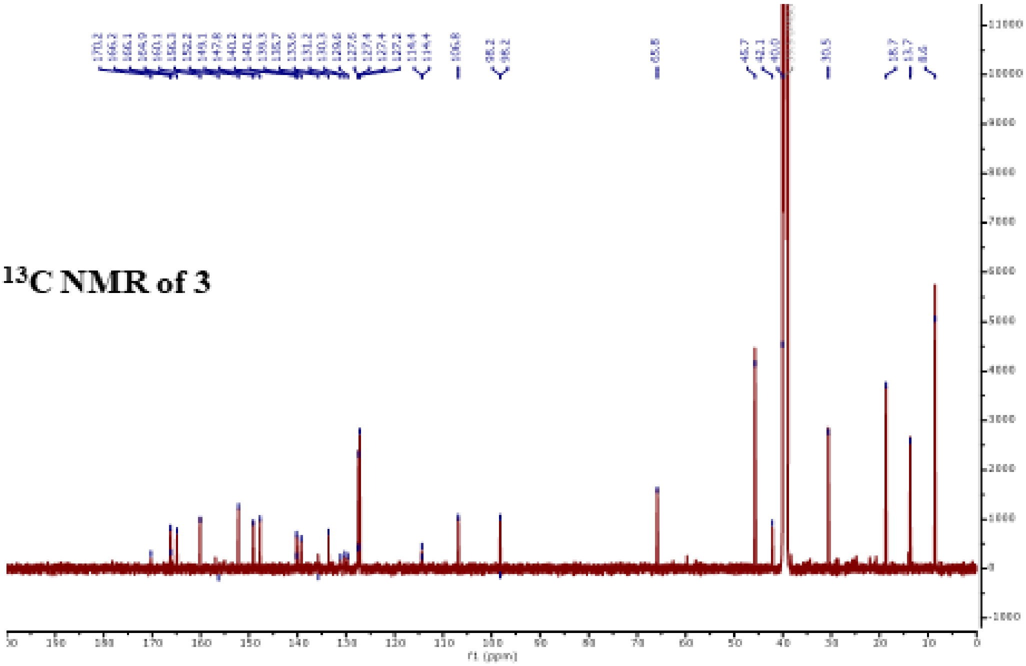

Supplementary Figure 7. NMR spectra of 3F-AF488 conjugate (compound 3 in Figure 4A)

^{1}H NMR (600 MHz, DMSO-d_6) δ 13.43 (s, 1H), 9.98 (s, 1H), 9.20 (s, 2H), 8.98 (s, 1H), 8.68 (s, 1H), 8.60 (s, 1H), 8.35 (s, 1H), 8.32 (d, J = 8.5 Hz, 1H), 8.25 (d, J = 8.0 Hz, 1H), 7.82 (d, J = 8.3 Hz, 2H), 7.47 (d, J = 7.7 Hz, 1H), 7.36 (d, J = 8.4 Hz, 2H), 7.00 (d, J = 7.7 Hz, 1H), 6.91 (s, 4H), 6.46 (s, 2H), 4.90 (s, 2H), 4.12 (t, J = 6.6 Hz, 2H), 3.56 – 3.46 (m, 4H), 3.10 (q, J = 7.2 Hz, 6H), 1.60 (dt, J = 14.4, 6.7 Hz, 2H), 1.35 (dq, J = 14.8, 7.4 Hz, 2H), 1.17 (t, J = 7.3 Hz, 9H), 0.88 (t, J = 7.4 Hz, 3H).

^{13}C NMR (151 MHz, DMSO-d_6) δ 170.2, 166.2, 166.1, 164.9, 160.1, 156.3, 152.2, 149.1, 147.8, 140.2, 140.2, 139.3, 135.7, 133.6, 131.2, 130.3, 129.6, 127.6, 127.4, 127.4, 127.2, 114.4, 114.4, 106.8, 98.2, 65.8, 45.7, 42.1, 40.0, 30.5, 18.7, 13.7, 8.6.

+ESI-HRMS m/z: calc'd for [M-H] $C_{40}H_{36}N_9O_{13}S_2^-$ =914.1879, found $C_{40}H_{36}N_9O_{13}S_2^-$=914.1894.

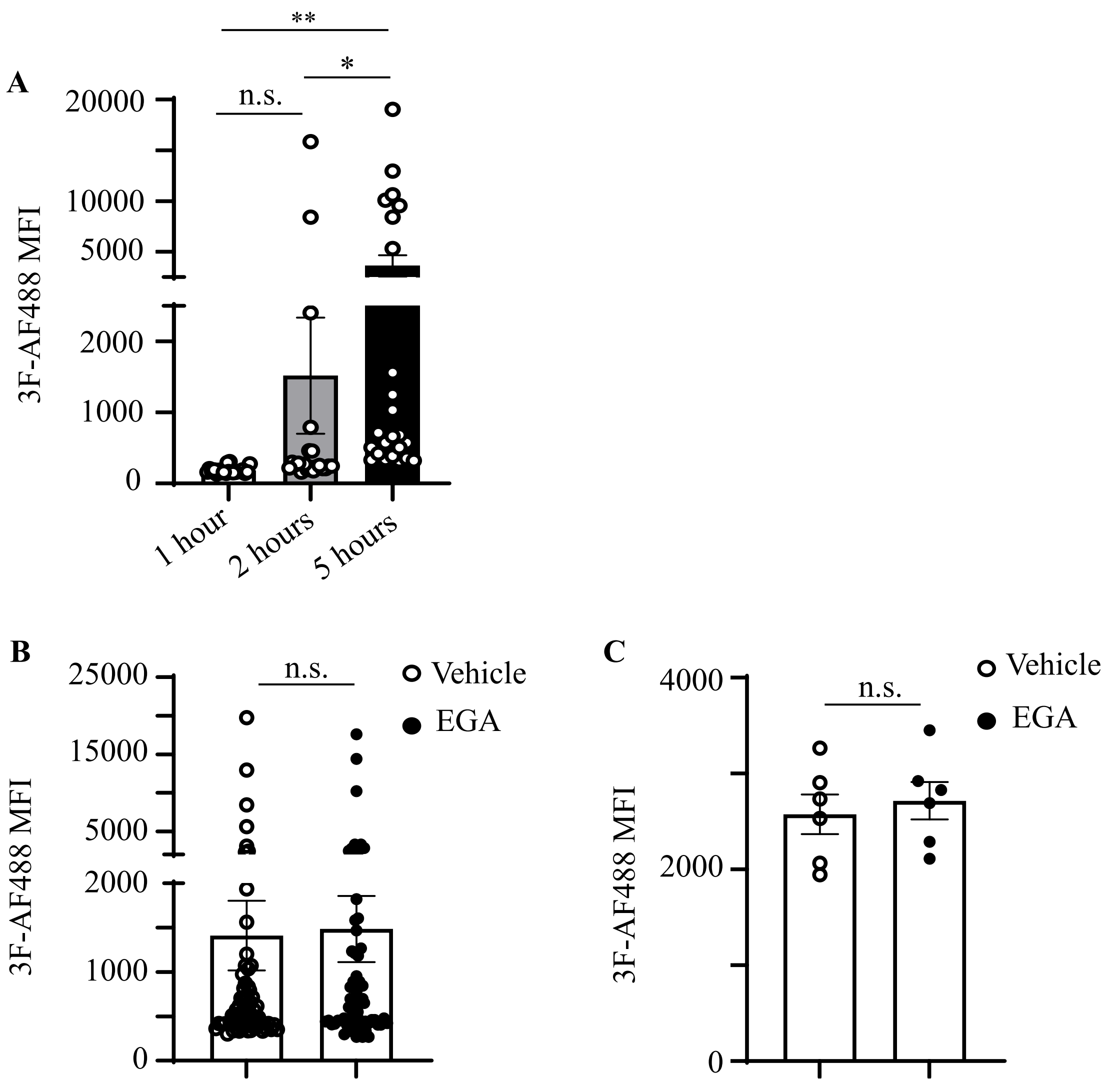

Supplementary Figure 8. Kinetics of 3F-AF488 conjugate uptakes by pDCs.

pDCs were incubated for 1, 2, and 5 hours in the presence of 20 µM 3F-AF488 conjugates. pDC uptakes of 3F-AF488 was measured with confocal microscopy (A). pDCs were pre-incubated with 20 µM EGA or vehicle, and then stimulated with 3F-AF488 conjugates for 5 hours. Fluorescence of 3F-AF488 conjugate was analyzed by confocal microscopy (B) as well as by flow cytometry (C). Data analyzed by one-way ANOVA. n.s.: non-significant, *p <0.05, and **p <0.01.

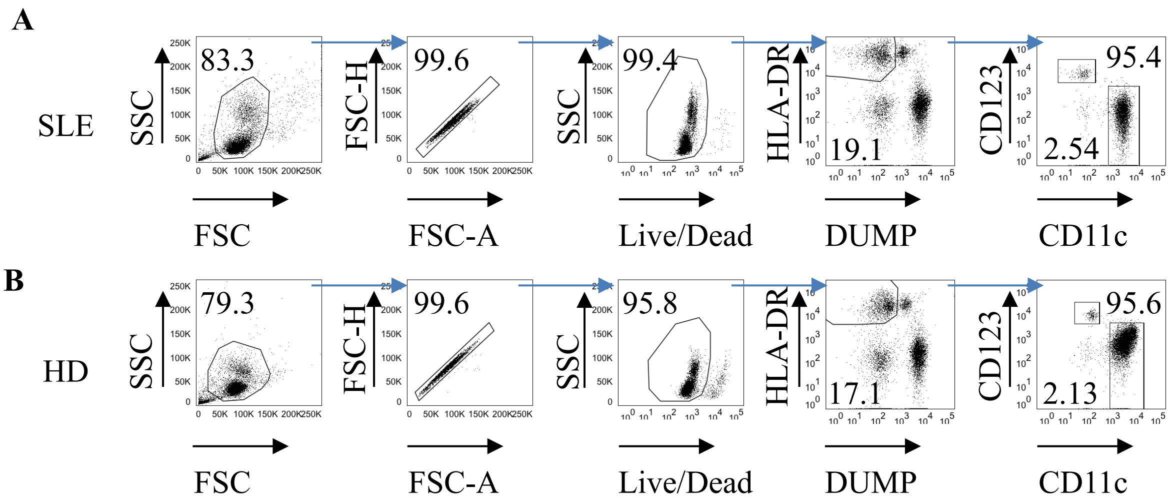

Supplementary Figure 9. Gating strategy for analysis of intracellular cytokine expression in pDCs and mDCs/monocytes.

PBMC cultures from systemic lupus erythematosus (SLE) patients (A) and healthy donors (HD) (B) were gated based on live singlet cells. Cells were further discriminated based on the expression of HLA-DR and DUMP (CD3, CD19, CD56) and then pDCs ($CD123^{+}CD11c^{-}$) and mDCs/monocytes ($CD123^{-}CD11c^{+}$) were gated.

Supplementary Table 1. Antibodies utilized in flow cytometry in this study.

Antibody	Clone	Source	Catalog
Anti-Human HLA-DR	L243	Biolegend	307618
Anti-Human HLA-DR	G46-6	BD	560651, 561359
Anti-Human Lineage-1	Various	BD, Biolegend	340546, 363601
Anti-Human CD3	UCHT1	BD, Biolegend	555535, 300412
Anti-Human CD3	Sk7	BD	340440
Anti-Human CD14	MϕP9	BD	557831
Anti-Human CD19	HIB19	BD	555415
Anti-Human CD20	2H7	eBioscience	17-0209-42
Anti-Human CD56	B159	BD	555518
Anti-Human CD123	9F5	BD	551065, 563161
Anti-Human CD123	7G3	BD	560826
Anti-Human CD11c	B-ly6	BD	560369, 561355, 562393
Anti-Human CD86	2331	BD	563412
Anti-Human IFNα	LT27:295	Miltenyi Biotec	130-092-601, 130-123-708
Anti-Human TNFα	Mab11	Biolegend	502932, 502936
Anti-Human IL-6	MQ2-13A5	eBioscience	46-7069-42
Mouse IgG1 Isotype	MOPC-21	Biolegend	400114, 400158, 400162

Supplementary Table 2. Antibodies utilized in immunoblotting in this study.

Antibody	Catalog	Host	Source	Dilution
Anti - ATF3	18665	Rabbit	CST	1:1000
Anti - p-p65 (S536)	3033	Rabbit	CST	1:1000
Anti - p65	6956	Mouse	CST	1:1000
Anti - p-IKKα/β (S176/S177)	2078	Rabbit	CST	1:1000
Anti - Iκβa	4812	Rabbit	CST	1:1000
Anti - p-p38 (T180/Y182)	9215	Rabbit	CST	1:1000
Anti - p38	9212	Rabbit	CST	1:1000
Anti - pSTAT1 (Y701)	9167	Rabbit	CST	1:1000
Anti – STAT1	14994	Rabbit	CST	1:1000
Anti - IRF7	4920	Rabbit	CST	1:1000
HRP - Anti - Mouse IgG	7076	Horse	CST	1:1000
HRP - Anti - Rabbit IgG	7074	Goat	CST	1:1000

Supplementary Table 3. Antibodies utilized in confocal microscopy in this study.

Antibody	Clone	Catalog #	Host	Source	Dilution
Anti-EEA1	14	610456	Mouse	BD	1:250
Anti-VAMP3	N/A	ab200657	Rabbit	Abcam	1:500
Anti-LAMP1	N/A	ab24170	Rabbit	Abcam	1:1000
Anti-LAMP2	H4B4	354301	Mouse	Biolegend	1:500
Rabbit Polyclonal IgG	N/A	LS-C149375	Rabbit	LSBio	1:5000
Mouse IgG1	MOPC-21	400102	Mouse	Biolegend	1:500
AF568-Goat-Anti-Rabbit IgG	N/A	A11036	Goat	ThermoFisher	1:1000
AF647-Goat-Anti-Mouse IgG	N/A	A21235	Goat	ThermoFisher	1:1000

Supplementary Table 4. Information of SLE patients recruited in this study.

Pt #	Age	Gender	Ethnicity	SLEDAI - 2k	Clinical Status
1	40	Female	White	4	1
2	56	Female	White	8	2
3	49	Female	White	10	2
4	59	Female	White	8	2
5	39	Female	White	8	2
6	44	Female	White	12	2
7	57	Male	White	12	2
8	63	Female	White	8	2
9	37	Female	White	0	2
10	50	Male	Pacific Islander	7	2

Clinical Status

1: No disease activity, off medications

2: Disease in remission, on medication

3: Disease, relapsing

4: Treatment resistant
